# A Note on Wavelet-Based Estimator of the Hurst Parameter

**DOI:** 10.3390/e22030349

**Published:** 2020-03-18

**Authors:** Liang Wu

**Affiliations:** Center of Statistical Research, School of Statistics, Southwestern University of Finance and Economics, Chengdu 611130, China; wuliang@swufe.edu.cn

**Keywords:** wavelet analysis, Hurst parameter, fractional Brownian motion, long-range dependence

## Abstract

The signals in numerous fields usually have scaling behaviors (long-range dependence and self-similarity) which is characterized by the Hurst parameter *H*. Fractal Brownian motion (FBM) plays an important role in modeling signals with self-similarity and long-range dependence. Wavelet analysis is a common method for signal processing, and has been used for estimation of Hurst parameter. This paper conducts a detailed numerical simulation study in the case of FBM on the selection of parameters and the empirical bias in the wavelet-based estimator which have not been studied comprehensively in previous studies, especially for the empirical bias. The results show that the empirical bias is due to the initialization errors caused by discrete sampling, and is not related to simulation methods. When choosing an appropriate orthogonal compact supported wavelet, the empirical bias is almost not related to the inaccurate bias correction caused by correlations of wavelet coefficients. The latter two causes are studied via comparison of estimators and comparison of simulation methods. These results could be a reference for future studies and applications in the scaling behavior of signals. Some preliminary results of this study have provided a reference for my previous studies.

## 1. Introduction

The signals in numerous fields usually have scaling behavior (long-range dependence and self-similarity) which has been recognized as a key property for data characterization and decision making (see e.g., [1,2,3,4,5]). It is usually characterized by the Hurst parameter *H* [6]. The key point for detecting the scaling behavior is the estimation of the Hurst parameter *H*. The Hurst parameter was first computed via R/S statistic by Hurst [7] for the study of hydrological properties of Nile river. Hurst found that R/S statistic on the Nile data grew approximately as $n^{H},\;H=0.74$. *n* is the number of observations. This phenomenon is called the Hurst phenomenon. To study the Hurst phenomenon, Mandelbrot introduced the concept of self-similar and explained the Hurst phenomenon successfully using self-similar fractional Brownian motion (FBM) [8]. A continuous process X(t) is said to be self-similar, if for a>0, $X\left(at\right)\overset{d}{=}a^{H}X\left(t\right)$, *H* is the self-similar parameter. When H>0.5, the increments of FBM are long-range dependent, i.e., the summation of their auto-covariances is divergent. Thus, fractal Brownian motion and its increments (fractional Gaussian noise (FGN)) play important roles in modeling signals with self-similarity and long-range dependence. Most studies on this issue are based on FBM.

Wavelet analysis is a common method for signal processing (see e.g., [9,10]) and has been widely used for the fractal analysis of signals due to its multiresolution. Nicolis et al. [11] defined three kinds of wavelet-based entropy for studying the two-dimensional fractional Brownian field. Li et al. [12] used wavelet fractal and twin support vector machine to study the classification of heart sound signals. Ramírez-Pacheco et al. [13] studied fractal signal classification using non-extensive wavelet-based entropy.

The wavelet-based estimator of the Hurst parameter was well-established by Abry et al. (see [14,15,16,17,18,19,20,21]). Compared with other estimators, such as the R/S method, the periodogram, the variogram (semi-parametric or nonparametric estimator) and the parametric method, the wavelet-based estimator performs well in both the statistical and computational sense, and is superior in robustness (see [18,19] and the references therein). Besides, the wavelet-based method can also eliminate some trends (linear trends, polynomial trend, or more) by the property of its vanishing moments [17], which makes the estimator robust to some nonstationarities. More simulation studies for the estimation of Hurst parameter can be seen in [22]. Based on the standard wavelet-based estimator, some robust estimators are proposed. Soltani et al. [23] proposed an improved wavelet-based estimator via the average of two wavelet coefficients of half length apart and taking the logarithm first. Shen et al. [24] proposed a robust estimator of self-similar parameter using wavelet transform, which was less sensitive to some non-stationary traffic conditions. Park & Park [25] introduced a robust wavelet-based estimator which took the logarithm of wavelet coefficients first and averaged them later. Feng & Vidakovic [26] estimated the Hurst parameter via a general trimean estimator on nondecimated wavelet coefficients Kang & Vidakovic [27] proposed a robust estimator of Hurst parameter via medians of log-squared nondecimated wavelet coefficients.

Despite extensive studies of standard wavelet-based estimator proposed by Abry et al., there is still a lack of comprehensive and detailed numerical simulation study on fractal Brownian motion, especially for the selection of parameters and the empirical bias. I have not seen studies on the changes of bias and variance with all different *H*s and with different data lengths, which I think is important for the selection of the lower octave bound $j_{1}$, especially at small values of *H*. $j_{1}$ is selected via the minimum mean square error. Thus, this paper conducts a detailed numerical simulation study on the selection of parameters including the following contents.

- The changes of bias and variance with all different *H*s, different data lengths, different $j_{1}$s and different wavelets;
- The relations of selected $j_{1}$ with data length and *H*;

For the causes of the empirical bias which exist in standard wavelet-based estimator, the following three causes in the case of FBM are concluded.

- The initialization for initial approximation wavelet coefficients which introduces errors in used detailed wavelet coefficients.
- The inaccurate bias correction caused by correlations of wavelet coefficients.
- The method of simulation for FBM is not enough exact that the empirical bias is caused.

There exist many studies on the reduction of empirical bias caused by the first two reasons, but lack of study on determining which is the main cause of empirical bias. It is important for reducing empirical bias via appropriate techniques. Combining with results of parameters selection, this paper analyzes the above three causes of empirical bias in the case of FBM via comparison of estimators and comparison of simulation methods. The results obtained from above numerical simulations can be a reference for future studies and applications in the scaling behavior of signals. Some preliminary results of this study have provided a reference for my previous studies on wavelet-based estimation of Hurst parameters [28,29,30].

This paper is organized as follows. In Section 2, this paper introduces two available estimators for the Hurst parameter, and the initialization methods for the initial approximation wavelet coefficients. The simulation methods of FBM are described in Section 3. The main results are reported and discussed in Section 4 and my works are concluded in Section 5.

## 2. Wavelet-Based Estimator

### 2.1. Definitions and Properties

Fractional Brownian motion {X(t),t∈R} with Hurst parameter H>0 is a real-valued mean-zero Gaussian process with the following covariance structure:

(1) $$\begin{array}{c} EX\left(t\right)X\left(s\right)=\frac{1}{2}{\left\{|t\right|}^{2H}+{\left|s\right|}^{2H}{-|t-s|}^{2H}\}. \end{array}$$

It is a self-similar process with stationary increment. Its wavelet coefficient is defined by

(2) $$\begin{array}{c} d_{X}\left(j,k\right)=\int_{R}\psi_{j,k}\left(t\right)X\left(t\right)dt. \end{array}$$

ψ(t) is the mother wavelet, which is defined through a scaling function ϕ(t). Usually we choose ψ(t) as a base function, and we can change it to $\psi_{j,k}\left(t\right)=2^{-j/2}\psi \left(2^{-j}t-k\right),j,k\in Z$. The factors $2^{j}$ and *j* are called the scale and octave respectively.

Please note that FBM is usually denoted by $B^{H}$. this paper uses the symbol *X* instead of $B^{H}$ since the methods in this section for FBM can be applicable to a more general process named self-similar process with stationary increment and finite variance [20].

Some key properties of the wavelet coefficient of *X* are given in the following lemma. The proof of this lemma can be found in [19,20,31,32,33].

**Lemma** **1.** *Let {X(t),t∈R} be a fractional Brownian motion. $\psi \left(t\right)\in L^{2}\left(R\right)$ is an orthonormal wavelet with compact support and have N≥1 vanishing moments. The wavelet coefficients of X(t) given in (2) have these properties below,*

*(1) $Ed_{X}\left(j,k\right)=0$ and $d_{X}\left(j,k\right)$ is Gaussian, for any j,k∈Z.*

*(2) For fixed j∈Z,*

(3) $$\begin{array}{c} d_{X}\left(j,k\right)\overset{d}{=}2^{j(H+1/2)}d_{X}\left(0,k\right),\;\forall k\in Z. \end{array}$$

*(3) For fixed j∈Z,*

(4) $$\begin{array}{c} d_{X}\left(j,k+h\right)\overset{d}{=}d_{X}\left(j,h\right),\;\forall k,h\in Z. \end{array}$$

*(4) For $j,j^{\prime },k,k^{\prime }\in Z$,*

(5) $$\begin{array}{c} Ed_{X}\left(j,k\right)d_{X}\left(j^{\prime },k^{\prime }\right)\approx |2^{j}k-2^{j^{\prime }}k^{\prime }{|}^{2H-2N},\;\left|2^{j}k-2^{j^{\prime }}k^{\prime }\right|\to +\infty . \end{array}$$

*In the above, $\overset{d}{=}$ means equality in distribution.*

**Remark** **1.** *In view of Equation (5), to avoid long-range dependence for $d_{X}\left(j,k\right)$, i.e., to ensure that $\sum_{j,k\in Z}E|d_{X}\left(j,k\right)d_{X}\left(0,0\right)|<\infty$, one needs to choose*

(6) $$\begin{array}{c} N>H+1/2,i.e.,\;(2H-2N)<-1, \end{array}$$

*that is, have at least N=2. Under this condition, the correlation of $d_{X}\left(j,k\right)$ tends rapidly to 0 at large lags.*

According to Remark 1 and Equation (5), let the number of vanishing moments N≥2. It is reasonable to impose the following assumptions.

- For fixed *j*, the $d_{X}\left(j,\cdot \right)$ are independent and identically distributed;
- The processes $d_{X}\left(j,\cdot \right)$ and $d_{X}\left(j^{\prime },\cdot \right)$, $j\neq j^{\prime }$, are independent.

Under these two assumptions, according to Lemma 1, there exist two available least squares estimators for the Hurst parameter. [19,20,34], one of which is first applied in the case of FBM for the bias study of common estimator.

### 2.2. Two Wavelet-Based Estimators

#### **The First Estimator** 

The first estimator is the standard wavelet-based estimator of Hurst parameter which is proposed by Abry et al and commonly used in applications of various fields. In view of Equations (3) and (4), I can check the following formula.

(7) $$\begin{array}{c} Ed_{X}^{2}\left(j,k\right)=C_{1}2^{j(2H+1)},\;C_{1}=Ed_{X}^{2}\left(0,0\right). \end{array}$$

Take the logarithm:

(8) $$\begin{array}{c} \log_{2}Ed_{X}^{2}\left(j,k\right)=j\left(2H+1\right)+\log_{2}C_{1}. \end{array}$$

So the estimation of *H* can be conducted by a linear regression in the left part versus *j* diagram. The $Ed_{X}^{2}\left(j,k\right)$ is estimated by

(9) $$\begin{array}{c} S\left(j\right):=1/n_{j}\sum d_{X}^{2}\left(j,k\right). \end{array}$$

where $n_{j}$ stands for the number of $d_{X}^{2}\left(j,k\right)$ actually available at octave *j*.

Due to different variances of $\log_{2}S\left(j\right)$ at different *j*s, the weighted least squares for this regression model is needed. The weight is the reciprocal of the variance of $\log_{2}S\left(j\right)$.

Please note that

(10) $$\begin{array}{c} E\log_{2}S\left(j\right)\neq \log_{2}ES\left(j\right)=\log_{2}Ed_{X}^{2}\left(j,k\right). \end{array}$$

This can lead to the bias of estimator.

Define the variables $y_{1}\left(j\right)$s as

$$\begin{array}{c} y_{1}\left(j\right):=\log_{2}S\left(j\right)-g\left(j\right). \end{array}$$

where g(j) is calculated such that $Ey_{1}\left(j\right)=\log_{2}ES\left(j\right)$. To ensure the unbiasedness of the estimator, I use $y_{1}\left(j\right)$ as the response variable instead of $\log_{2}S\left(j\right)$. Moreover, $\mathrm{Var}y_{1}\left(j\right)=\mathrm{Var}\log_{2}S\left(j\right)$.

The calculation of g(j) and $\mathrm{Var}y_{1}\left(j\right)$ are shown in [18,19,20].

(11) $$\begin{array}{cc} \; & g\left(j\right)=\Gamma^{\prime }\left(n_{j}/2\right)/\left(\Gamma \left(n_{j}/2\right)\ln2\right)-\log_{2}\left(n_{j}/2\right), \end{array}$$

(12) $$\begin{array}{cc} \; & \mathrm{Var}y_{1}\left(j\right)=\varsigma \left(2,n_{j}/2\right)/\ln^{2}2, \end{array}$$

where $\varsigma \left(2,z\right):=\sum_{x=0}^{\infty }1/{\left(z+x\right)}^{2}$ is a generalized Riemann Zeta function. Γ and $\Gamma^{\prime }$ are the gamma function and its derivative respectively.

The g(j) and $\mathrm{Var}y_{1}\left(j\right)$ can be also calculated via sample moment estimators [18,19,20]

(13) $$\begin{array}{cc} \; & g\left(j\right)\approx -\frac{\log_{2}e}{2}C\left(j\right), \end{array}$$

(14) $$\begin{array}{cc} \; & \mathrm{Var}y_{1}\left(j\right)\approx {\left(\log_{2}e\right)}^{2}C\left(j\right), \end{array}$$

where $C\left(j\right)=\mathrm{Var}d_{X}^{2}\left(j,\cdot \right)/\left[n_{j}{\left(Ed_{X}^{2}\left(j,\cdot \right)\right)}^{2}\right]$. The C(j) term is estimated using the sample moment estimators of the fourth and second moments of $d_{X}\left(j,\cdot \right)$ at each octave.

#### **The Second Estimator** 

As mentioned above, since $E\log_{2}S\left(j\right)\neq \log_{2}ES\left(j\right)$, the first estimator needs to correct bias. For avoiding this case, the second least squares estimator for Hurst parameter is proposed. This estimator is also originally proposed by Abry et al. [34] and then studied by Park & Park [25] for the purpose of robustness.

Based on Equations (3) and (4),

$$d_{X}^{2}\left(j,k\right)\overset{d}{=}2^{j(2H+1)}d_{X}^{2}\left(0,0\right).$$

Now take the logarithm first and then the expectation, obtain the following new equation.

(15) $$\begin{array}{c} E\log_{2}d_{X}^{2}\left(j,k\right)=j\left(2H+1\right)+C_{2},\;C_{2}=E\log_{2}d_{X}^{2}\left(0,0\right). \end{array}$$

So the estimation of *H* can be conducted by a weighted linear regression in the left part versus *j* diagram. The $E\log_{2}d_{X}^{2}\left(j,k\right)$ is estimated by

(16) $$\begin{array}{c} LS\left(j\right):=1/n_{j}\sum \log_{2}d_{X}^{2}\left(j,k\right). \end{array}$$

where $n_{j}$ stands for the number of $d_{X}^{2}\left(j,k\right)$ actually available at octave *j*.

Compared with the first estimator, the second estimator changes the order of expectation and logarithmic. The idea of this estimator is first proposed for analyzing the α-stable self-similar processes with infinite second-order statistics and long-range dependence [34].

Define the variables $y_{2}\left(j\right)$ as

$$\begin{array}{c} y_{2}\left(j\right):=LS\left(j\right). \end{array}$$

We can check that $Ey_{2}\left(j\right)=ELS\left(j\right)=E\log_{2}d_{X}^{2}\left(j,k\right)$. Let $y_{2}\left(j\right)$ be the response variable of weighted linear regression. The unbiasedness of the estimator follows from the unbiasedness of $y_{2}\left(j\right)$.

Similar to the calculation shown in [18,19,20], the variance of $y_{2}\left(j\right)$ can be calculated for the weight of regression.

(17) $$\begin{array}{c} \mathrm{Var}y_{2}\left(j\right)=\varsigma \left(2,1/2\right)/\left(n_{j}\ln^{2}2\right), \end{array}$$

And by sample moment estimators [18,19,20]

(18) $$\begin{array}{c} \mathrm{Var}y_{2}\left(j\right)=\mathrm{Var}\log_{2}d_{X}^{2}\left(j,\cdot \right)/n_{j}. \end{array}$$

The $\mathrm{Var}\log_{2}d_{X}^{2}\left(j,\cdot \right)$ is estimated using its sample variance at each octave.

#### **Explicit Formula of theTwo Estimators** 

Let $j_{1}$ denote the lower bound of *j*, and $j_{2}$ denote the upper bound of *j*, i.e., the values of *j* are chosen $j_{1}\leq j\leq j_{2}$. According to the weighted least squares, the explicit formula of estimators can be obtained as follows,

(19) $$\begin{array}{c} \hat{H}=\frac{\sum_{j=j_{1}}^{j_{2}}\omega (j)y(j)-1}{2}, \end{array}$$

where $\omega \left(j\right)=\frac{T_{0}j-T_{1}}{\sigma^{2}\left(j\right)\left(T_{0}T_{2}-T_{1}^{2}\right)}$, $T_{0}=\sum_{j=j_{1}}^{j_{2}}1/\sigma^{2}\left(j\right)$, $T_{1}=\sum_{j=j_{1}}^{j_{2}}j/\sigma^{2}\left(j\right)$, $T_{2}=\sum_{j=j_{1}}^{j_{2}}j^{2}/\sigma^{2}\left(j\right)$.

When using the first method, $y\left(j\right)=y_{1}\left(j\right)$ and $\sigma^{2}\left(j\right)=\mathrm{Var}y_{1}\left(j\right)$, let $\hat{H_{1}}$ denote the first estimator. When using the second method, $y\left(j\right)=y_{2}\left(j\right)$ and $\sigma^{2}\left(j\right)=\mathrm{Var}y_{2}\left(j\right)$, let $\hat{H_{2}}$ denote the second estimator.

#### **Variance Comparison** 

The variances of $\hat{H_{1}}$ and $\hat{H_{2}}$ can be compared via a simple theoretical analysis. In view of Equation (19), the variance of $\hat{H}$ can be calculated by

(20) $$\begin{array}{c} \mathrm{Var}\hat{H}=\frac{1}{4}\sum_{j=j_{1}}^{j_{2}}\omega^{2}\left(j\right)\sigma^{2}\left(j\right). \end{array}$$

When $n_{j}$ is large, recall that the asymptotic form of $\mathrm{Var}y_{1}\left(j\right)$ (see [19]).

(21) $$\begin{array}{c} \mathrm{Var}y_{1}\left(j\right)∼2/\left(n_{j}\ln^{2}2\right). \end{array}$$

Also recall Equation (17),

$$\begin{array}{c} \mathrm{Var}y_{2}\left(j\right)=\varsigma \left(2,1/2\right)/\left(n_{j}\ln^{2}2\right). \end{array}$$

So when $n_{j}$ is large, the asymptotic form of ratio can be obtained,

(22) $$\begin{array}{c} \frac{\mathrm{Var}\hat{H_{2}}}{\mathrm{Var}\hat{H_{1}}}∼\frac{\varsigma (2,1/2)}{2}=2.47. \end{array}$$

The variance of $\hat{H_{1}}$ is smaller than that of $\hat{H_{2}}$.

Please note that the nondecimated wavelet coefficients have been used in wavelet-based estimator since they can decrease the variance due to their redundancy [26,27]. However, they can also increase the correlations in wavelet coefficients. Then when using nondecimated wavelet coefficients, we should take logarithm first. It is suitable to reduce the variance of the second estimator via nondecimated wavelet coefficients. For further considering the possible outliers caused by logarithmic transform, Kang & Vidakovic [27] suggest using medians for estimation of Hurst parameter in this case. This method is denoted by MEDL.

### 2.3. Calculation of Wavelet Coefficients

According to the multiresolution analysis (MRA), the wavelet coefficients can be calculated by fast pyramidal algorithm. The scaling function ϕ and the wavelet ψ satisfy so-called two-scale equation:

(23) $$\begin{array}{cc} \; & \phi \left(t/2\right)=\sqrt{2}\sum_{n}u_{n}\phi \left(t-n\right), \end{array}$$

(24) $$\begin{array}{cc} \; & \psi \left(t/2\right)=\sqrt{2}\sum_{n}v_{n}\phi \left(t-n\right), \end{array}$$

where $\left\{u_{n}\right\}$ and $\left\{v_{n}\right\}$ are two existing sequences belonging to $l^{2}$.

Define the approximation coefficients $a_{X}\left(j,k\right)$:

$$\begin{array}{c} a_{X}\left(j,k\right):=\int_{R}\phi_{j,k}\left(t\right)X\left(t\right)dt \end{array}$$

where $\phi_{j,k}\left(t\right)=2^{-j/2}\phi \left(2^{-j}t-k\right)$.

So $d_{X}\left(j,k\right)$ can be calculated by fast pyramidal algorithm.

(25) $$\begin{array}{c} a_{X}\left(j,k\right)=\sum_{n}u_{n}a_{X}\left(j-1,2k+n\right), \end{array}$$

(26) $$\begin{array}{c} d_{X}\left(j,k\right)=\sum_{n}v_{n}a_{X}\left(j-1,2k+n\right). \end{array}$$

In view of above formulas, the $a_{X}\left(0,\cdot \right)$ are obtained via integral. However, in practice, the data we obtained are always discrete and finite. The $a_{X}\left(0,\cdot \right)$ cannot be obtained by integral in continuous time. When sampling frequency is high and the scale of wavelet transform is small, the typical approach is to set [35,36,37]

(27) $$\begin{array}{c} a_{X}\left(0,k\right)=X\left(k\right). \end{array}$$

where {X(k),k∈Z,1≤k≤n} is discrete and finite FBM.

In view of Equations (25) and (26), the number of available wavelet coefficients $n_{j}$ decreases by half. Then $n_{j}\approx n2^{-j}$.

**Remark** **2.** *For a wavelet which has time support (finite or decreases very fast as |t|→∞), an increase in the number of vanishing moments N comes with an enlargement of the time support [20]. In the case of finite data, because of the boundary effects of wavelet transform, this will lead to the decrease of the number of available wavelet coefficients $n_{j}$ at each octave.*

### 2.4. The Initialization Method

The discrete sampling for a continuous process X(t) usually implies an irrevocable loss of information on X(t) [35]. So the approach shown in Equation (27) introduces errors in $d_{X}\left(j,k\right)$s. It is known [14,18] that these initialization errors are significant on small octaves but quickly decrease with increasing *j*. For large *j*, the initialization issue can be ignored. Veitch et al. [35] introduce an initialization method for discrete time series, which has been proved meaningful for correction of the initialization errors in the case of long-range dependent process.

This initialization method is based on the stochastic version of the Shannon sampling theorem [35,38]. Consider the bandlimited stationary stochastic process {X(t),t∈R}, construct $\tilde{X}\left(t\right)$ by

(28) $$\begin{array}{c} \tilde{X}\left(t\right)=\sum_{n=-\infty }^{\infty }X(n)\mathrm{sinc}(t-n),\;\mathrm{where}\;\mathrm{sinc}(t)=\frac{\sin\pi t}{\pi t}. \end{array}$$

The $\tilde{X}\left(t\right)$ is bandlimited, and has the same spectral density as that of X(t) in the frequency band [−1/2,1/2] (otherwise is zero). It is easy to check

$$\begin{array}{c} \left\{\tilde{X}\left(k\right),k\in Z\right\}=\left\{X\left(k\right),k\in Z\right\}. \end{array}$$

Furthermore,

(29) $$\begin{array}{cc} a_{X}\left(0,k\right) & =\int_{R}\phi \left(t-k\right)\tilde{X}\left(t\right)dt\\ \; & =\sum_{n=-\infty }^{\infty }X\left(n\right)\int_{R}\phi (t-k)\mathrm{sinc}(t-n)dt\\ \; & =\sum_{n=-\infty }^{\infty }X(n)I(k-n), \end{array}$$

where $I\left(m\right)=\int_{R}\phi (t)\mathrm{sinc}(t+m)dt$. The sequence {I(m)} is calculated in [35].

Please note that because of the boundary effects, the initialization will lead to the decrease of the number of available wavelet coefficients $n_{j}$.

## 3. Simulation of FBM

For studying the statistical performance of the two estimators in the case of FBM, the numerical simulation of FBM is conducted. Here, this section briefly introduces two simulation methods of FBM [39,40,41,42]. Let {X(t),t∈[0,1]} be a mean-zero fractional Brownian motion with Hurst parameter H∈(0,1).

### ***The Cholesky Method*** 

The Cholesky method uses the Cholesky decomposition of the covariance matrix. The FBM generated by this method is exact in the sense of covariance structure, but this method is slow.

Let $\Sigma =(\Sigma_{i,j})$ be the covariance matrix of FBM, where $\Sigma_{i,j}=\mathrm{Cov}\left(X\left(t_{i}\right),X\left(t_{j}\right)\right)$, $t_{i}=i/n,t_{j}=j/n,i,j=1,\ldots ,n$. Conduct the Cholesky decomposition $\Sigma =AA^{\prime }$.

At last, $X={\left(X\left(t_{1}\right),\cdots ,X\left(t_{n}\right)\right)}^{\prime }=AZ$ is the generated FBM, where $Z={\left(Z_{1},\cdots ,Z_{n}\right)}^{\prime }$, $Z_{1},\cdots ,Z_{n}$ are independent and identically distributed N(0,1).

### ***The Circulant Embedding Method*** 

The simulation procedure is based on the method of circulant embedding. The algorithm of circulant embedding was originally proposed by Davies and Harte [39], and was later simultaneously generalized by Dietrich and Newsam (see [40,41,42] and the references therein). It has been regarded as a fast and exact simulation of stationary Gaussian processes [42]. I use this method to generate a fractional Gaussian noise, and construct a fractional Brownian motion via the cumulative sum of generated fractional Gaussian noise [41].

First consider the fractional Gaussian noise, which is a zero-mean stationary Gaussian process $\left\{Z_{k},k=1,\ldots n\right\}$ with covariance

(30) $$\begin{array}{c} \mathrm{Cov}\left(Z_{k},Z_{k+\Delta k}\right)=\frac{{\left|\Delta k+1\right|}^{2H}{+|\Delta k-1|}^{2H}-2{\left|\Delta k\right|}^{2H}}{2},\;\Delta k=0,\ldots ,n. \end{array}$$

Such a stationary Gaussian noise can be efficiently and exactly generated by the method of circulant embedding and fast Fourier transform [41,42]. The fractional Brownian motion {X(t),t∈[0,1]} is constructed on a uniformly spaced grid via the cumulative sum [41]

(31) $$\begin{array}{c} X\left(t_{k}\right)=n^{-H}\sum_{i=1}^{k}Z_{i},\;k=1,\ldots ,n. \end{array}$$

## 4. Simulation Results and Discussions

This section focuses on the numerical study of commonly used wavelet-based estimator (the first estimator) which still lacks of comprehensive and detailed numerical study on estimation of fractal Brownian motion, especially on its empirical bias and the selection of parameters. The second estimator was also compared with the commonly used estimator in this section for the purpose of empirical bias analysis. If not specified, the sample trajectory of FBM used in this section is generated by the circulant embedding method.

### 4.1. Selection of Parameters

It is a key step to select octaves *j*s and the number of vanishing moments *N* (or wavelet) before estimation. First this subsection studies the selection of these parameters for later estimations. For octaves *j*s, the lower bound $j_{1}$ and the upper bound $j_{2}$ need to be determined. The $j_{2}$ is chosen as the largest possible. In practice, it is set equal to

$$\begin{array}{c} j_{2}=\left\lfloor \log_{2}n-C\right\rfloor , \end{array}$$

where *n* denotes the data length and *C* is a constant (with value $\log_{2}\left(2N+1\right)$ corresponding to the length of the support of the wavelet [20]). As the discussion in Section 2, the initialization for $a_{X}\left(0,k\right)$ given in (27) introduces errors in the $d_{X}\left(j,k\right)$. It is known [14,18] that initialization errors are significant on small octaves but decrease with increasing *j*. So small octave cannot be chosen as $j_{1}$. Based on prior studies [18,20], and this paper selects $j_{1}$ by the minimum mean square error (MSE), where the MSE is defined as

$$\begin{array}{c} \mathrm{MSE}\left(\hat{H}\right):=E{\left(\hat{H}-H\right)}^{2}={\left(E\hat{H}-H\right)}^{2}+\mathrm{var}\left(\hat{H}\right). \end{array}$$

**Figure 1:**
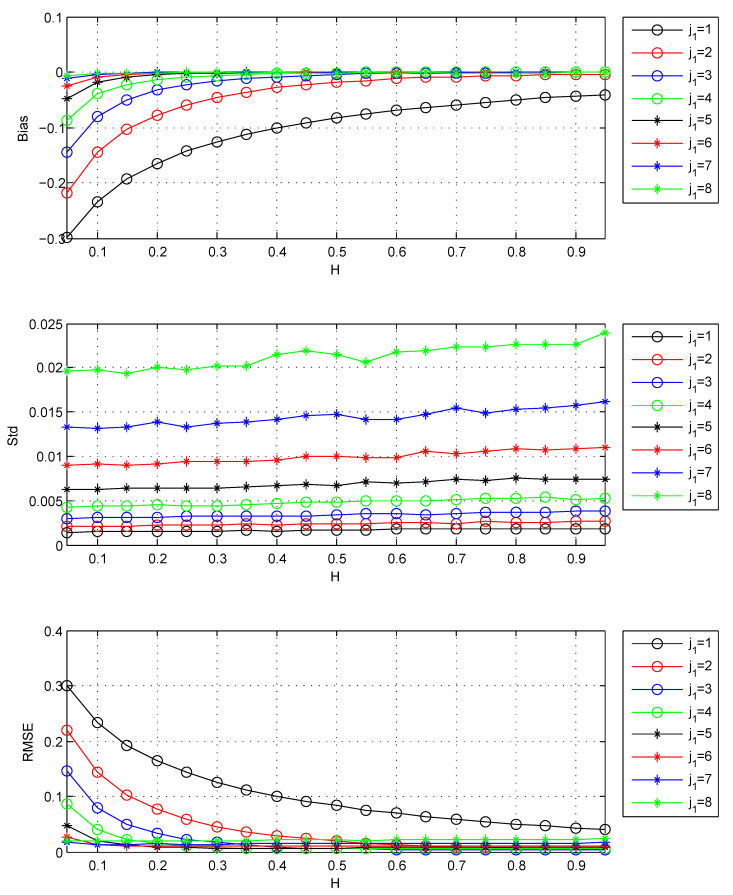
The Bias, Std, RMSE for estimators: $j_{1}$ is the lower bound of octaves *j*s. Std is the standard deviation, Bias $=E\hat{H}-H$, RMSE is the square root of MSE. The values of Std, Bias, and RMSE are the estimated versions of those for 1000 independent copies of FBM with length $n=2^{18}$. The used wavelet is the Daubechies wavelet with N=3 vanishing moments.

It allows the tradeoff between variance and bias. The results for the selection of $j_{1}$ are shown in Table 1 and Figure 1.

Figure 1 shows that the increase of $j_{1}$ causes the decrease of bias and the increase of standard deviation for all *H*s. So it is suitable to choose the $j_{1}$ by the minimum of MSE. From Table 1, when H>0.5, $j_{1}$ is chosen $j_{1}=3$ by minimum MSE. When 0.4≤H≤0.5, the RMSE of $j_{1}=3$ is close to that of chosen $j_{1}=4$. So considering most *H*s, $j_{1}=3$ should be chosen in the case of FBM.

**Figure 2:**
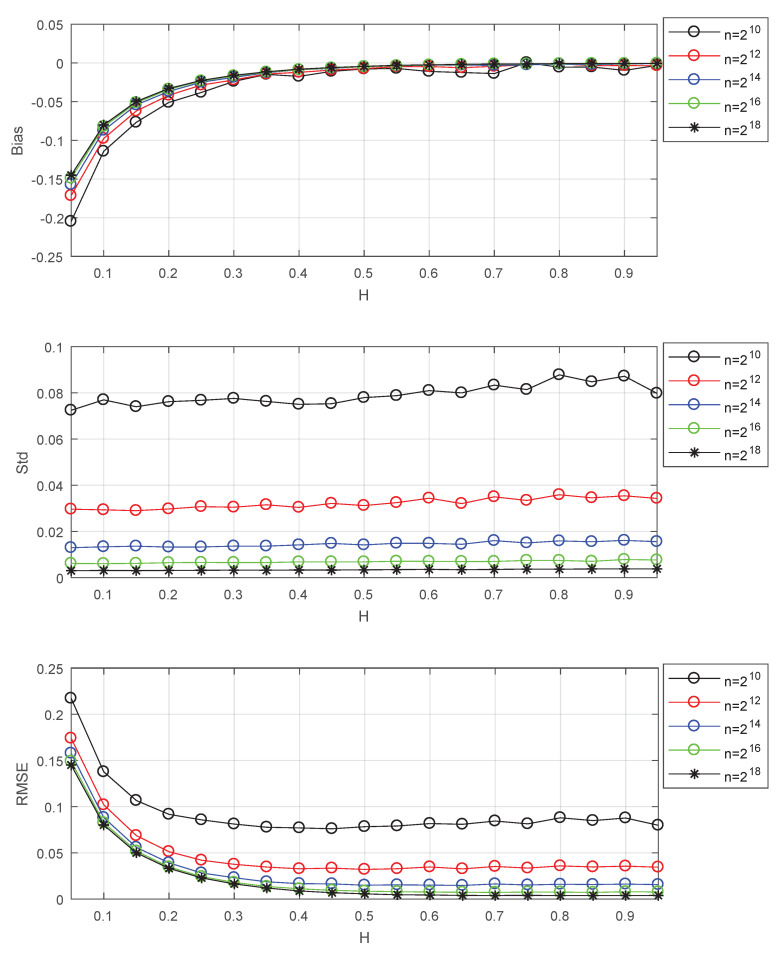
The Bias, Std, RMSE for estimators: *n* is the data length. Std is the standard deviation, Bias $=E\hat{H}-H$, RMSE is the square root of MSE. The values of Std, Bias, and RMSE are the estimated versions of those for 1000 independent copies of FBM with length *n*. The lower bound of octaves *j*s is chosen $j_{1}=3$. The used wavelet is the Daubechies wavelet with N=3 vanishing moments.

Please note that the results of Figure 1 and Table 1 are based on long series. In this case, the variances of all *H*s are small. For small values of *H*, the bias is large, and the MSE is mainly determined by the bias. So the estimator of small *H* trends to select large $j_{1}$ which can lead to small bias. Now I study the effects of data length and the selection of $j_{1}$ at different data lengths. The results of this issue are shown in Figure 2 and Table 2.

Figure 2 shows that the data length has little effect on the bias, but its decrease causes the increase of standard deviation for all *H*s. The increase of standard deviation may affect the selection of $j_{1}$. Thus, continue to use the minimum MSE to select $j_{1}$ at different data lengths for the tradeoff between variance and bias. The results of selection are shown in Table 2. From Table 2, it can be seen that the selected $j_{1}$ increases with the increase of data length, and the smaller the value of *H*, the faster the increase. Based on simulation results, the following formula is given for explanation.

$$\begin{array}{c} \min_{j_{1}}\mathrm{MSE}\left(\hat{H}\right)=\min_{j_{1}}\left[{\mathrm{Bias}}^{2}\left(H,j_{1}\right)+\mathrm{var}\left(j_{1},n\right)\right], \end{array}$$

where $\mathrm{Bias}(H,j_{1})$ denotes the bias of estimator which decreases with the increase of *H* and the increase of $j_{1}$. $\mathrm{var}(j_{1},n)$ denotes the variance which decreases with the decrease of $j_{1}$ and the increase of *n*. When *n* increases, the variance becomes smaller, the selected $j_{1}$ trends to increase for the tradeoff between variance and bias. The smaller the value of *H*, the larger the bias, and the more the selected $j_{1}$ increase.

**Figure 3:**
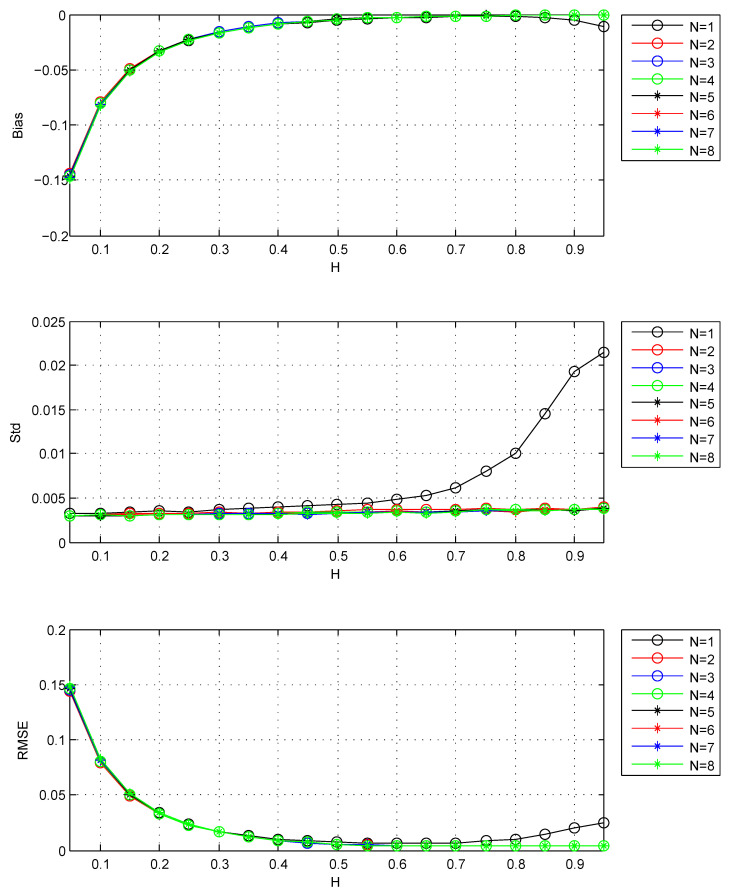
The Bias, Std, RMSE for estimators: *N* is the number of vanishing moments of Daubechies wavelet. Std is the standard deviation, Bias $=E\hat{H}-H$, RMSE is the square root of MSE. The values of Std, Bias and RMSE are the estimated versions of those for 1000 independent copies of FBM with length $n=2^{18}$. The lower bound of octaves *j*s is chosen $j_{1}=3$.

For the wavelet, this paper chooses the classical Daubechies wavelets, which are orthonormal and have compact support. According to Remark 1, the number of vanishing moments must be chosen N≥2. For analyzing the effect of *N*, I use N=1∼8 to estimate the Hurst parameter of FBM. The results are shown in Figure 3.

From Figure 3, when N≥2, the increase of *N* makes no improvements to the performance of the estimator. Besides, according to Remark 2, large *N* will cause the loss of available wavelet coefficients. So appropriately we should choose N=3.

**Figure 4:**
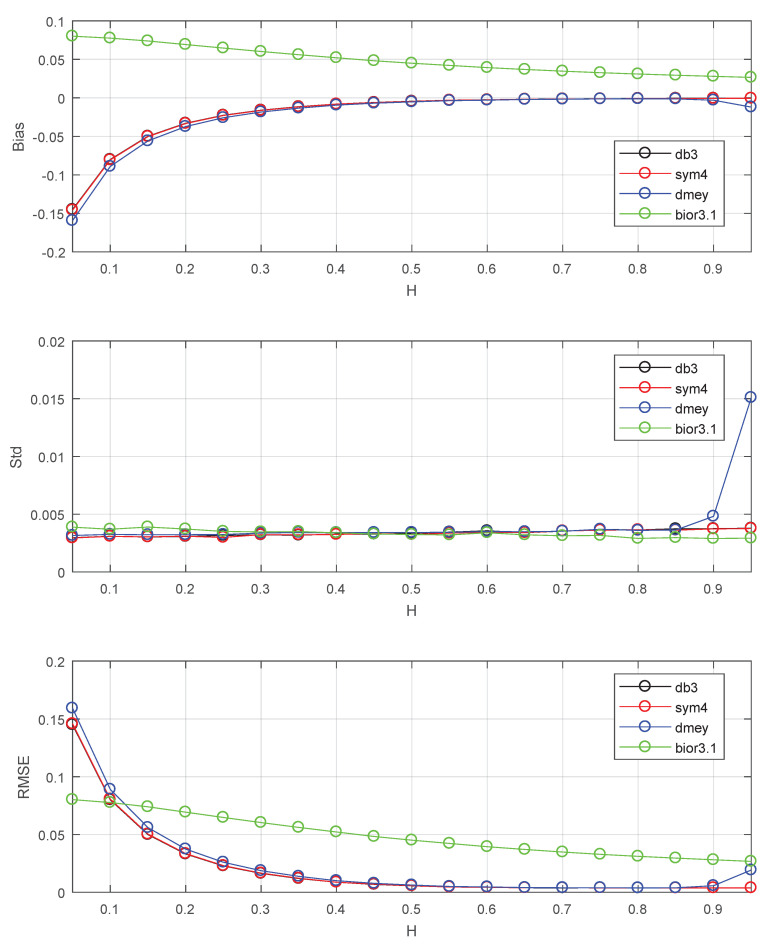
The Bias, Std, RMSE for estimators: db3 stands for Daubechies wavelet with three vanishing moments, sym4 stands for Symlets wavelet with four vanishing moments, dmey stands for discrete Meyer wavelet, bior3.1 stands for biorthogonal spline wavelets with orders $N_{r}=3$ (vanishing moments) and $N_{d}=1$. The values of Std, Bias and RMSE are the estimated versions of those for 1000 independent copies of FBM with length $n=2^{18}$. The lower bound of octaves *j*s is chosen $j_{1}=3$.

Finally, this subsection studies the performance of this estimator using various wavelets for further chosen of wavelet. The results are shown in Figure 4. db3 stands for Daubechies wavelet with three vanishing moments. sym4 stands for Symlets wavelet with four vanishing moments. dmey stands for discrete Meyer wavelet. bior3.1 stands for biorthogonal spline wavelets with orders $N_{r}=3$ (vanishing moments) and $N_{d}=1$. Since the Symlets wavelet with three vanishing moments has the same filters as db3, this part uses this kind of wavelet with four vanishing moments. The first three wavelets are orthogonal and have compact support. The last wavelet is biorthogonal. It can be seen in Figure 4 that performance using the first three wavelets are almost the same except for the standard deviation of dmey at H=0.95. The biorthogonal spline wavelet performs worse than orthogonal wavelets except at H≤0.1. This is due to the large bias caused by strong correlations of biorthogonal wavelet coefficients, and is consistent with the conclusion of Lemma 1. We need to use orthogonal compact supported wavelet to control these correlations via vanishing moments.

### 4.2. Results and Discussions on Empirical Bias

This subsection conducts a detailed numerical analysis on the empirical bias exits in the commonly used wavelet-based estimator (the first estimator). Based on previous analysis, the following three possible causes of empirical bias are concluded.

- The initialization for $a_{X}\left(0,k\right)$ given in (27) introduces errors in $d_{X}\left(j,k\right)$, and the initialization errors are significant on small octaves but decrease with increasing *j*.
- The inaccurate bias correction for $E\log_{2}S\left(j\right)\neq \log_{2}ES\left(j\right)$ (under independent assumptions) caused by correlations of wavelet coefficients.
- The method of simulation for FBM is not enough exact that the empirical bias is caused.

From results of Section 4.1, I have the following information on empirical bias

- The increase of *N* and change of wavelet made no improvements to the empirical bias. The chosen of biorthogonal wavelet makes the empirical bias worse.
- The increase of $j_{1}$ leads to the decrease of empirical bias.
- The empirical bias increases with the decrease of *H*. when choosing $j_{1}=3$ and N=3, the empirical bias of estimator $\hat{H_{1}}$ can be ignored for H≥0.4. So the estimator $\hat{H_{1}}$ is suitable to detect the long-range dependence (can be described by H>0.5).

The fact that increase of $j_{1}$ leads to decrease of empirical bias is consistent with the first cause. As we know, the larger the value of *H* is, the smoother the sample path of FBM is, and the more exact the initialization given in (27) is. It is consistent with the fact that the empirical bias increases with the decrease of *H*. So I conclude that the initialization errors caused by (27) contribute to the empirical bias.

The first information indicates the empirical bias is related to correlations of wavelet coefficients. However, this effects can be fixed (maybe eliminated) via the selection of orthogonal compact supported wavelet.

Next, after choosing the orthogonal compact supported wavelet (db3) and fixing $j_{1}=3$, this study analyzes the latter two causes via comparing with the second estimator and comparison of simulation methods respectively.

#### **Comparison of Estimators** 

**Figure 5:**
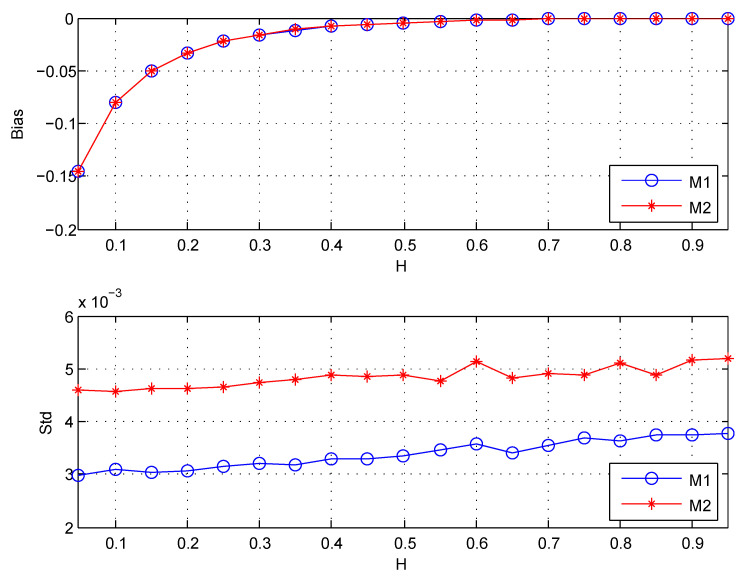
The Bias and Std for estimators: M1 denotes the first estimator, M2 denotes the second estimator. Std is the standard deviation, Bias $=E\hat{H}-H$. The values of Std and Bias are the estimated versions of those for 1000 independent copies of FBM with length $n=2^{18}$.

Since the unbiasedness of the second estimator $\hat{H_{2}}$ is get naturally without independence assumptions of wavelet coefficients. This part compares $\hat{H_{2}}$ with $\hat{H_{1}}$ for studying its empirical bias. The length of simulation data is $n=2^{18}$. $\left(j_{1},j_{2}\right)$ are chosen (3,15). The wavelet coefficients are computed using the classical Daubechies wavelet with N=3 vanishing moments. The results are shown in Figure 5 and Table 3.

Table 3 shows the results for the estimator of ratio given in Equation (22). It indicates that the variance of $\hat{H_{2}}$ is about twice that of $\hat{H_{1}}$, which roughly satisfy the theoretical results given in (22). From Figure 5, it can be seen that when H<0.4, both $\hat{H_{1}}$ and $\hat{H_{2}}$ have the same obvious bias despite the theoretical unbiasedness of the two estimators under independence assumptions of wavelet coefficients. Besides, the same as the results shown in Table 3, the standard deviation (Std) of $\hat{H_{2}}$ is larger than that of $\hat{H_{1}}$.

Because the empirical bias also exists in $\hat{H_{2}}$ whose unbiasedness is get naturally, and is the same as that of $\hat{H_{2}}$. I conclude that the empirical bias of $\hat{H_{1}}$ is not due to the inaccurate bias correction for $E\log_{2}S\left(j\right)\neq \log_{2}ES\left(j\right)$ caused by correlations of wavelet coefficients.

Besides, considering the variances of the two estimators, we should choose the first estimator $\hat{H_{1}}$ for the estimation of Hurst parameter.

#### **Comparison of Simulation Methods** 

**Figure 6:**
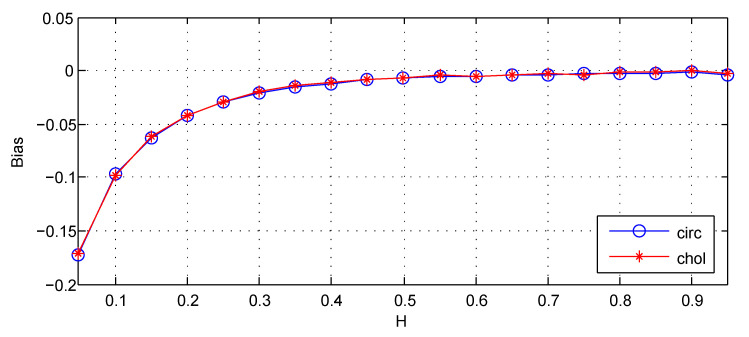
The Bias for estimators: circ denotes the circulant embedding method for simulation of FBM, chol denotes the Cholesky method for simulation of FBM. Bias $=E\hat{H}-H$. The values of Bias are the estimated versions of those for 1000 independent copies of FBM with length $n=2^{12}$. The lower bound of octaves *j*s is chosen $j_{1}=3$. The used wavelet is the Daubechies wavelet with N=3 vanishing moments.

For the third cause, this part applies $\hat{H_{1}}$ to the FBM that is exactly generated by the Cholesky method for comparison. The results are shown in Figure 6.

From Figure 6, it can be seen that estimations for the FBM respectively generated by the Cholesky method and the circulant embedding method has almost the same empirical bias. I conclude that the method of simulation is not the cause of empirical bias.

### 4.3. Analysis of the Initialization Method

It has been shown above that the empirical bias of $\hat{H_{1}}$ is due to the initialization errors caused by (27). The initialization method given in (29) has proved effective for errors in the case of long-range dependent process [35]. Although FBM is not a bandlimited stationary stochastic process, I tend to check whether this method is suitable for FBM.

**Figure 7:**
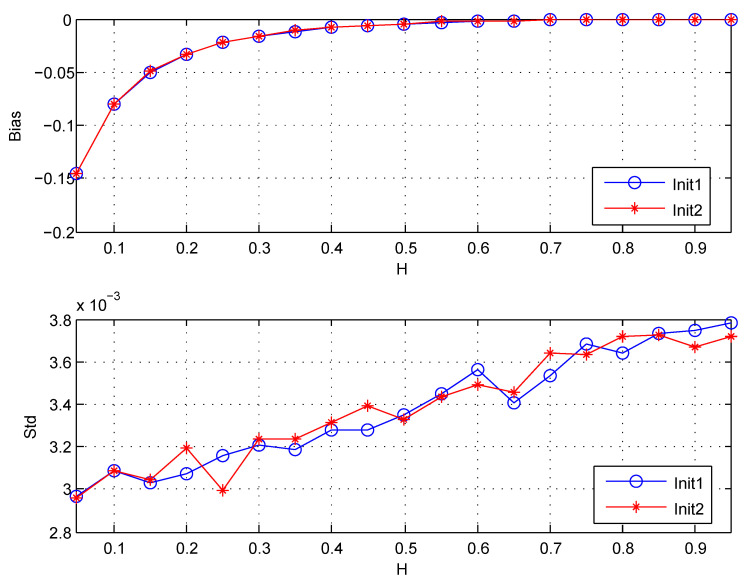
The Bias and Std for estimators: Init1 denotes the initialization by itself (or by (27)), Init2 denotes the initialization denoted by (29). Std is the standard deviation, Bias $=E\hat{H}-H$. The values of Std and Bias are the estimated versions of those for 1000 independent copies of FBM with length $n=2^{18}$.

This subsection applies the estimator $\hat{H_{1}}$ with this initialization to FBM for analysis, and compares it with the initialization by itself (or by Equation (27)). The length of simulation data is $n=2^{18}$. $\left(j_{1},j_{2}\right)$ are chosen (3,15). The wavelet coefficients are computed using the classical Daubechies wavelet with N=3 vanishing moments. The results are shown in Figure 7.

Figure 7 shows that both Biases and Stds for the two initializations are almost the same. It indicates that the initialization method given in (29) is inefficient in the case of FBM. Beside, it is known that the method Init2 will lead to the decrease of the number of available wavelet coefficients $n_{j}$ for the boundary effects, which may result in the increase of the variance of estimator. So I suggest choosing the initialization for $a_{X}\left(0,k\right)$ given in (27) in the future work.

### 4.4. Analysis of Noise Effects

At last, this paper adds this subsection for analysis of noise effects on the first estimator, which possibly happen in the real data. Various independent and identically distributed noises are added to the generated FBM for this issue. The signal-to-noise ratio (SNR) is defined as follows.

$$\begin{array}{c} SNR=\sqrt{\frac{\mathrm{var}X(1)}{\mathrm{var}\varepsilon }}, \end{array}$$

where ε means noise. Set SNR=2 in this subsection.

**Figure 8:**
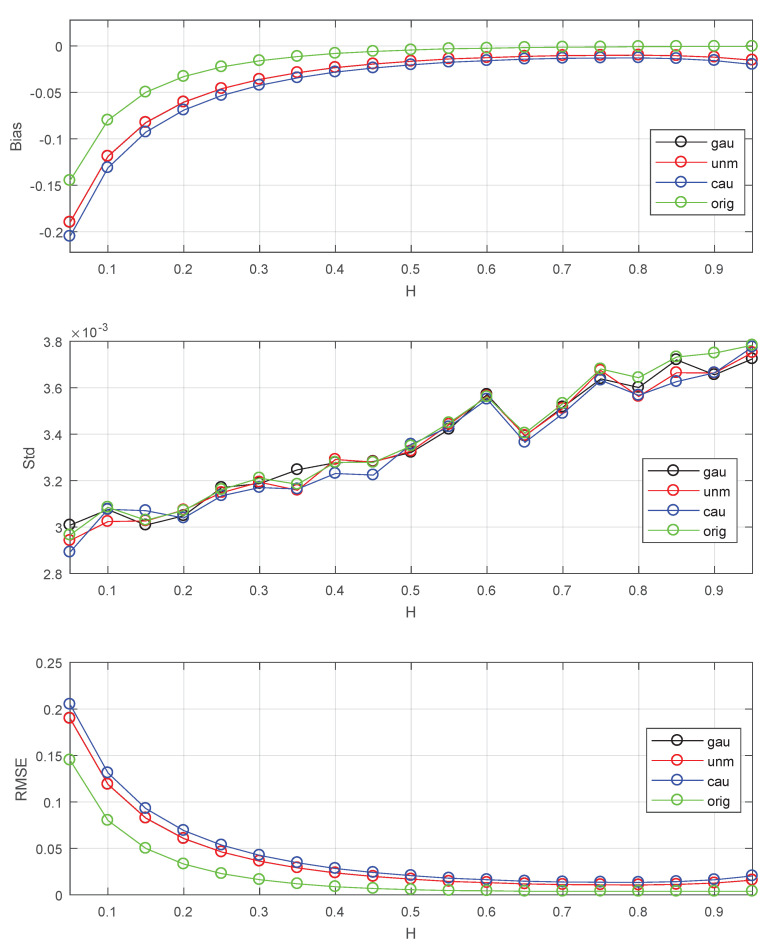
The Bias, Std, RMSE for estimators: orig stands for original series without noise, gau stands for Gaussian noise, unm stands for uniform noise, cau stands for Cauchy noise. The values of Std, Bias and RMSE are the estimated versions of those for 1000 independent copies of FBM with noise. The data length $n=2^{18}$. The lower bound of octaves *j*s is chosen $j_{1}=3$. The used wavelet is the Daubechies wavelet with N=3 vanishing moments

The results are shown in Figure 8. From Figure 8, I found that noises have some effects on the performance, and can lead to increase of bias. The effects of Gaussian and uniform noises are almost the same. The Cauchy noise can cause more increase of bias than the other two noises.

## 5. Conclusions

This paper focuses on the numerical simulation study of wavelet-based estimators in the case of FBM concluding the selection of parameters and the analysis of empirical bias which have not been studied comprehensively in previous studies. This study adds to previous numerical simulation studies of wavelet-based estimators which are not comprehensive in the case of FBM.

Results of the parameter selection showed that the increase of lower bound $j_{1}$ causes the decrease of bias and the increase of standard deviation for all *H*s, and suggested $j_{1}=3$ via the minimum mean square error at a long data length $n=2^{18}$. In addition, it was also found that the empirical bias increased with the decrease of *H* and could be ignored for H≥0.4 when $j_{1}=3$ and N=3. The effects of *n* on performance and relations of selected $j_{1}$ with *n* were also concluded via simulation studies. It was shown that the data length had little effect on the bias, but its decrease caused the increase of standard deviation for all *H*s. The selected $j_{1}$ increased with the increase of data length, and the smaller the value of *H*, the faster the increase. For the vanishing moments *N*, when N≥2, the increase of *N* made no improvements to the performance of estimator. The change of orthogonal wavelets made no improvements to the empirical bias. The chosen of biorthogonal wavelet made empirical bias worse.

The analysis of empirical bias was conducted first via comparison of two available estimators and comparison of simulation methods. The results showed that the empirical bias was due to the initialization errors caused by discrete sampling, and was not related to simulation methods. When choosing an appropriate orthogonal compact supported wavelet, the empirical bias was almost not related to the inaccurate bias correction caused by correlations of wavelet coefficients. I regret to note that the initialization method given in (29), which has proved effective in the case of long-range dependent process, made no improvements to the empirical bias. All these results will be a guide for my future studies.

## Figures and Tables

**Table 1:** Estimation quality for FBM series. On the left, the $j_{1}$ for minimum MSE and its Bias, Std, RMSE is given. On the right, the same quantities with $j_{1}=3$ are also given for comparison. RMSE is the square root of MSE. All the results are the estimated versions of Bias, Std, RMSE for 1000 independent copies of FBM with length $n=2^{18}$. The used wavelet is the Daubechies wavelet with N=3 vanishing moments.

*H*	$j_{1}^{\mathrm{MSE}}$	Bias	Std	RMSE	$j_{1}$	Bias	Std	RMSE
0.05	7	−0.0122	0.0133	0.0180	3	−0.1450	0.0030	0.1451
0.10	6	−0.0087	0.0091	0.0126	3	−0.0801	0.0031	0.0801
0.15	6	−0.0046	0.0090	0.0101	3	−0.0499	0.0030	0.0500
0.20	5	−0.0056	0.0064	0.0085	3	−0.0330	0.0031	0.0331
0.25	5	−0.0032	0.0063	0.0071	3	−0.0226	0.0032	0.0228
0.30	5	−0.0019	0.0063	0.0066	3	−0.0160	0.0032	0.0163
0.35	4	−0.0038	0.0045	0.0059	3	−0.0115	0.0032	0.0119
0.40	4	−0.0023	0.0047	0.0052	3	−0.0081	0.0033	0.0087
0.45	4	−0.0019	0.0048	0.0052	3	−0.0060	0.0033	0.0068
0.50	4	−0.0012	0.0048	0.0049	3	−0.0044	0.0033	0.0056
0.55	3	−0.0030	0.0034	0.0046	3	−0.0030	0.0034	0.0046
0.60	3	−0.0025	0.0036	0.0044	3	−0.0025	0.0036	0.0044
0.65	3	−0.0018	0.0034	0.0038	3	−0.0018	0.0034	0.0038
0.70	3	−0.0014	0.0035	0.0038	3	−0.0014	0.0035	0.0038
0.75	3	−0.0013	0.0037	0.0039	3	−0.0013	0.0037	0.0039
0.80	3	−0.0008	0.0036	0.0037	3	−0.0008	0.0036	0.0037
0.85	3	−0.0006	0.0037	0.0038	3	−0.0006	0.0037	0.0038
0.90	3	−0.0006	0.0037	0.0038	3	−0.0006	0.0037	0.0038
0.95	3	−0.0006	0.0038	0.0038	3	−0.0006	0.0038	0.0038

**Table 2:** Estimation quality for FBM series. On the left, the $j_{1}$ for minimum MSE and its Bias, Std, RMSE is given. On the right, the same quantities with $j_{1}=3$ are also given for comparison. RMSE is the square root of MSE. All the results are the estimated versions of Bias, Std, RMSE for 1000 independent copies of FBM. The used wavelet is the Daubechies wavelet with N=3 vanishing moments.

*H*	*n*	$j_{1}^{\mathrm{MSE}}$	Bias	Std	RMSE	$j_{1}$	Bias	Std	RMSE
	$2^{10}$	2	−0.0632	0.0473	0.0789	3	−0.0239	0.0776	0.0811
	$2^{12}$	3	−0.0220	0.0305	0.0376	3	−0.0220	0.0305	0.0376
0.3	$2^{14}$	4	−0.0080	0.0202	0.0217	3	−0.0186	0.0136	0.0231
	$2^{16}$	4	−0.0063	0.0096	0.0115	3	−0.0167	0.0065	0.0179
	$2^{18}$	5	−0.0019	0.0063	0.0066	3	−0.0160	0.0032	0.0163
	$2^{10}$	2	−0.0276	0.0479	0.0553	3	−0.0078	0.0779	0.0783
	$2^{12}$	2	−0.0231	0.0202	0.0307	3	−0.0073	0.0312	0.0320
0.5	$2^{14}$	3	−0.0048	0.0142	0.0149	3	−0.0048	0.0142	0.0149
	$2^{16}$	3	−0.0050	0.0068	0.0085	3	−0.0050	0.0068	0.0085
	$2^{18}$	4	−0.0012	0.0048	0.0049	3	−0.0044	0.0033	0.0056
	$2^{10}$	2	−0.0109	0.0526	0.0537	3	−0.0056	0.0878	0.0879
	$2^{12}$	2	−0.0061	0.0233	0.0241	3	−0.0010	0.0359	0.0359
0.8	$2^{14}$	2	−0.0062	0.0106	0.0123	3	−0.0015	0.0159	0.0160
	$2^{16}$	3	−0.0010	0.0074	0.0075	3	−0.0010	0.0074	0.0075
	$2^{18}$	3	−0.0008	0.0036	0.0037	3	−0.0008	0.0036	0.0037

**Table 3:** Estimations of ratio of variance.

*H*	0.05	0.10	0.15	0.20	0.25	0.30	0.35	0.40	0.45	0.50
$\hat{\mathrm{Var}}\hat{H_{2}}/\hat{\mathrm{Var}}\hat{H_{1}}$	2.41	2.18	2.32	2.27	2.16	2.19	2.27	2.22	2.18	2.13
*H*	0.55	0.60	0.65	0.70	0.75	0.80	0.85	0.90	0.95	
$\hat{\mathrm{Var}}\hat{H_{2}}/\hat{\mathrm{Var}}\hat{H_{1}}$	1.92	2.08	2.00	1.94	1.76	1.95	1.71	1.89	1.88	
